# PLAG1 deficiency impairs spermatogenesis and sperm motility in mice

**DOI:** 10.1038/s41598-017-05676-4

**Published:** 2017-07-13

**Authors:** Almas R. Juma, Sylvia V. H. Grommen, Moira K. O’Bryan, Anne E. O’Connor, D. Jo Merriner, Nathan E. Hall, Stephen R. Doyle, Pauliina E. Damdimopoulou, Daniel Barriga, Adam H. Hart, Wim J. M. Van de Ven, Bert De Groef

**Affiliations:** 1 Department of Physiology, Anatomy and Microbiology, La Trobe University, Bundoora, Victoria 3086 Australia; 2 Development and Stem Cells Program of Monash Biomedicine Discovery Institute, and Department of Anatomy and Developmental Biology, Monash University, Clayton, Victoria 3800 Australia; 3 Department of Biochemistry and Genetics and La Trobe Institute for Molecular Sciences, La Trobe University, Bundoora, Victoria 3086 Australia; 4 Department of Clinical Sciences, Intervention and Technology, Karolinska Institute, and Karolinska University Hospital, 14183 Huddinge, Sweden; 5 Department of Human Genetics, KU Leuven, B-3000 Leuven, Belgium; 6 Wellcome Trust Sanger Institute, Hinxton, Cambridge, CB10 1SA United Kingdom

## Abstract

Deficiency in pleomorphic adenoma gene 1 (PLAG1) leads to reduced fertility in male mice, but the mechanism by which PLAG1 contributes to reproduction is unknown. To investigate the involvement of PLAG1 in testicular function, we determined (i) the spatial distribution of PLAG1 in the testis using X-gal staining; (ii) transcriptomic consequences of PLAG1 deficiency in knock-out and heterozygous mice compared to wild-type mice using RNA-seq; and (iii) morphological and functional consequences of PLAG1 deficiency by determining testicular histology, daily sperm production and sperm motility in knock-out and wild-type mice. PLAG1 was sparsely expressed in germ cells and in Sertoli cells. Genes known to be involved in spermatogenesis were downregulated in the testes of knock-out mice, as well as *Hsd17b3*, which encodes a key enzyme in androgen biosynthesis. In the absence of *Plag1*, a number of genes involved in immune processes and epididymis-specific genes were upregulated in the testes. Finally, loss of PLAG1 resulted in significantly lowered daily sperm production, in reduced sperm motility, and in several animals, in sloughing of the germinal epithelium. Our results demonstrate that the subfertility seen in male PLAG1-deficient mice is, at least in part, the result of significantly reduced sperm output and sperm motility.

## Introduction

Infertility is a major health problem worldwide: the prevalence of infertility in developed and developing countries ranges from 3.5% to 16.7%^1^. In about 10–15% of couples, the cause of infertility is not found using routine diagnostic tests, and of the remaining cases, 45–50% show a male contribution to infertility^2^. In 30–45% of cases, abnormal semen is found, but the underlying cause is never identified^3, 4^.

In the first instance, fertility is reliant on the production of functional gametes. In the male, spermatogenesis entails the production of haploid gametes in the seminiferous tubules of the testes. Undifferentiated spermatogonia proliferate to produce type A and B spermatogonia via mitosis; type B spermatogonia differentiate to primary spermatocytes, which undergo meiosis to round spermatids. This involves coordinated regulation by thousands of genes^5, 6^. The round spermatids then go through spermiogenesis, which is the transformation into spermatozoa. During their development, germ cells are supported and nourished by Sertoli cells, somatic cells present along the basement membrane of the seminiferous tubule, extending inwards into the adluminal compartment. Each Sertoli cell envelopes 30–50 developing germ cells, as they differentiate and migrate inwards toward the lumen^5, 7, 8^. Testicular steroidogenesis on the other hand, takes place in the interstitial Leydig cells, and production of androgens is essential for male sexual differentiation as well as the maintenance of spermatogenesis^9^. Defects in either of these two testicular processes, spermatogenesis or steroidogenesis, can therefore lead to infertility.

Many cases of infertility of unknown cause are thought to have genetic aetiologies. Recent studies also indicate the involvement of genetic polymorphisms as risk factors for impairment of spermatogenesis^10^. One candidate gene is pleomorphic adenoma gene 1 (*PLAG1*), whose gene product, a zinc finger transcription factor, is best known for its involvement in the development of various human cancers via stimulation of cell proliferation^11, 12^. In addition, PLAG1 is believed to play a key role in regulating aspects of reproduction. Mice with targeted disruption of both alleles of *Plag1* (Gene ID: 56711) are characterised by reduced fertility in both sexes^13^. Wild-type (WT; *Plag1* ^+/+^) females had a conception rate of only 7% when mated with knock-out (KO; *Plag1* ^−/−^) males, which was only a third of the conception rate in WT × WT matings. Although KO females had normal conception rates when mated with WT males, their average litter size was reduced by half compared to WT females. Mating of both male and female KO mice resulted in infrequent conception and smaller litter sizes^13^. The cause of these fertility issues in *Plag1* KO mice is not known.

The aim of this study was to investigate the role of PLAG1 in testis function using WT, heterozygous (HET; *Plag1* ^+/−^), and KO mice. We present data demonstrating the spatial distribution of PLAG1 in the testes, the transcriptomic changes associated with the loss of one or two alleles of *Plag1* relative to WT mice, and the functional consequences of PLAG1 deficiency via histology of the testis, daily sperm production and sperm motility in male KO mice and age-matched WT mice, in order to characterise the reproductive abnormalities of PLAG1-deficient mice.

## Results

### PLAG1 is expressed in multiple cell types in the seminiferous tubules

**Figure 1:**
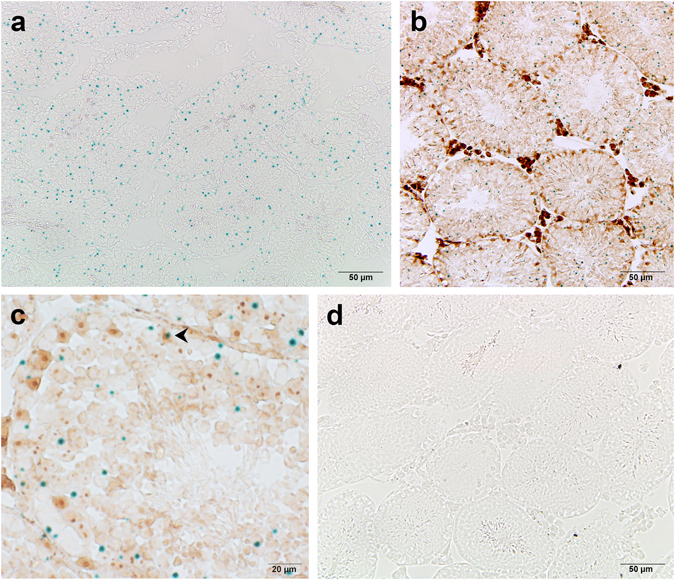
PLAG1-expressing cells in adult mouse testis. (**a**) *lacZ*-positive cells (blue) in a representative testis section of a 5-week-old *Plag1* knock-out animal, as determined by X-gal staining. (**b**,**c**) Expression of *lacZ* (blue; X-gal staining) was found mostly in germ cells and only occasionally (arrowhead) in Sertoli cells (brown, GATA4 immunostaining). (**d**) Wild-type negative control for X-gal staining, showing no blue signal.

In the *Plag1* KO mice, the entire *Plag1* coding sequence has been replaced by the *lacZ* reporter gene^13^, resulting in temporal and spatial expression of β-galactosidase in place of PLAG1. This model therefore allows the use of X-gal staining to investigate PLAG1 expression sites in KO and HET tissues. While X-gal staining was abundantly present in the pars distalis of the pituitary gland (Supplementary Fig. S1), known to express ample PLAG1^13^, sparse X-gal staining was observed across the seminiferous tubules in testes from KO mice (Fig. 1a,b). X-gal staining was found in spermatogonia, spermatocytes and spermatids, but within each tubule, only in a small number of cells. Likewise, immunohistochemical staining of GATA4, a Sertoli cell marker, combined with X-gal staining, showed only occasional *lacZ* expression in Sertoli cells (Fig. 1b,c). Virtually no staining occurred in the testicular interstitium of 5-week-old mice; in older mice, non-specific staining was observed that was also present in the WT negative control (Supplementary Fig. S2). Testis from HET animals showed the same expression pattern as that found in KO animals, but no staining was seen in testis from 5-week-old WT animals (‘negative control’) (Fig. 1d). WT animals never showed staining in the seminiferous tubules.

### Transcriptome analysis of *Plag1* WT, HET and KO mouse testis

**Figure 2:**
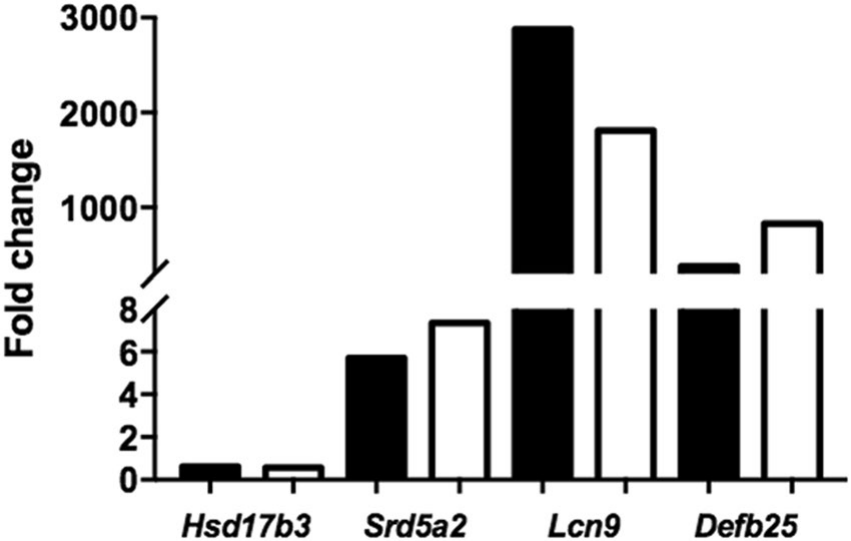
Change in mRNA expression levels of selected genes in the testes of *Plag1* knock-out compared to wild-type mice. White bars show the fold change values as determined by quantitative PCR; black bars show the fold change values as determined by RNA sequencing (*n* = 3). *Hsd17b3*, hydroxysteroid dehydrogenase 17 beta 3; *Srd5a2*, steroid reductase 5 alpha 2; *Lcn9*, lipocalin 9; *Defb25*, defensin beta 25.

RNA-seq was used to compare the transcriptomes of whole testes from 5-week-old *Plag1* WT, HET and KO mice, each performed in triplicate. Five-week-old animals were chosen to minimise the histological differences between WT and KO testes, and thus differences in gene expression as a direct consequence of differences in cell content. *Plag1* expression levels were verified in each of the individuals to confirm the genotypes. A false-discovery rate (FDR) below 0.05 was considered indicative of differential gene expression. Eighty-three genes were differentially expressed between HET and KO testes, compared to 377 differentially expressed genes between the WT and KO testes. There were 148 genes differentially expressed between the WT and HET testes. A comparison of all three genotypes revealed a total of 434 differentially expressed genes consisting of 248 upregulated and 186 downregulated genes in the KO testes (Supplementary Fig. S3). The top-15 upregulated and downregulated genes according to increasing FDR are listed in Tables 1 and 2, respectively. Gene ontology (GO) analysis using GOrilla and PANTHER indicated that most of the downregulated genes were involved in reproductive processes, fertilisation and meiosis, while the upregulated genes were involved in immune processes, as well as cell adhesion and angiogenesis (Supplementary Tables S1 and S2, Supplementary Fig. S4). Various germ cell markers (e.g. *Kit* and *Zbtb16* for spermatogonia, *Tex11* and *Klhl10* for premeiotic spermatocytes, *Sp10* for round spermatids, *Zpbp* and *Gapdhs* for mature sperm) were not differentially expressed, confirming that the KO and WT mice were capable of producing all germ cell stages (Supplementary Table S3). Several other markers were differentially expressed: eight Sertoli cell markers (*Ccnd2*, *Kitl, Espn*, *Mrc1*, *Frzb, Slc7a1, Daam2, Shbg, Mcf1*), nine Leydig cell markers (*Amy1, Fetub, Apoc1, Itih2, Lrg1, Insl3, 9230104L09Rik, Klk1b22, Slc38a1*) and two germ cell markers (*Stra8*, *Dazl*) (Supplementary Table S3). Overall, there was an upregulation of the Sertoli cell markers and a downregulation of the Leydig cell markers, with a few exceptions. The Sertoli marker *Kitl* was downregulated, while the rest were all upregulated. The Leydig markers *9230104L09Rik* and *Slc38a1* were upregulated, while the others were downregulated. Steroid 5 alpha-reductase 2 (*Srd5a2*) was upregulated, while sulfotransferase family 1E member 1 (*Sult1e1*) and hydroxysteroid 17-beta dehydrogenase 3 (*Hsd17b3*) were downregulated in the testes of KO mice (Supplementary Table S3). The genes encoding the follicle-stimulating hormone receptor (*Fshr*) and luteinising hormone receptor (*Lhr*) were not differentially expressed (Supplementary Table S3). Differential expression of selected genes (*Hsd17b3, Srd5a2, Lcn9, Def25b*) was confirmed with quantitative PCR (Fig. 2). For all genes tested, the average expression level in the KO testis as determined by qPCR was significantly different to that of the WT (*t*-test, *P* < 0.05), except for *Srd5a2* (*P* = 0.1534). Collectively, these changes in gene expression indicate that, overall, loss of PLAG1 expression leads to reduced expression of genes involved in spermatogenesis and testosterone synthesis, while genes involved in immune responses and epididymis-specific functions are upregulated (see Discussion).

**Table 1:** Top-15 upregulated genes in the testes of PLAG1*-*deficient mice.

Gene symbol	Gene name	FDR	Log fold change	Counts per million			GO terms		
				WT	HET	KO	Molecular function	Cellular component	Biological process
*Lcn9*	Lipocalin 9	5.62E-22	11.485	0.0	0.03	64.3	Catalytic activity, Binding	Extracellular region	Metabolic process, Cellular process, Localization
*4933400L20Rik*	RIKEN cDNA 4933400L20 gene	5.62E-22	1.614	8.9	27.2	28	ND	ND	ND
*Teddm1*	Transmembrane epididymal protein 1 A	1.26E-17	9.823	0.07	0.1	54.8	ND	Membrane	ND
*Lcn8*	Lipocalin 8	2.81E-16	10.196	0.07	0.03	92.8	Catalytic activity, Binding	Extracellular region	Metabolic process, Cellular process, Localization
*9230110F15Rik*	RIKEN cDNA 9230110F15 gene	2.32E-15	10.761	0.0	0.0	22.7	ND	ND	ND
*Ovch2*	Ovochymase 2	8.46E-15	9.763	0.0	0.03	11.2	Catalytic activity	Extracellular region	Reproduction, Metabolic process
*Adam28*	A disintegrin and metallopeptidase domain 28	1.84E-14	10.465	0.03	0.03	45.4	Catalytic activity, Binding	Membrane	Reproduction, Apoptotic process, Multicellular organismal process, Development process
*Gpx5*	Glutathione peroxidase 5	1.45E-12	12.180	0.1	32.2	499.7	Catalytic activity, Antioxidant activity	Extracellular region, Extracellular matrix, Membrane	Immune system process, Metabolic process, Response to stimulus
*5830403L16Rik (Teddm2)*	Transmembrane epididymal family member 2	2.89E-12	3.594	1.2	0.96	15.4	ND	ND	ND
*Defb25*	Defensin beta 25	7.70E-12	8.565	0.03	0.07	15.4	ND	Cell part, Extracellular region	Immune system process, Response to stimulus
*Gm4846*	Predicted gene 4846	3.65E-11	4.586	1.5	1.5	37.1	Catalytic activity	ND	Metabolic process
*Cst11*	Cystatin 11	3.30E-10	7.700	0.2	0.7	46.07	Catalytic activity, Binding, Enzyme regulator activity	Extracellular region	Metabolic process, Biological regulation
*Lcn10*	Lipocalin 10	5.86E-10	12.554	0.0	0.13	21.3	Catalytic activity, Binding	Extracellular region	Metabolic process, Cellular process, Localization
*Gm3428*	Predicted gene 3428	6.83E-10	8.739	0.0	0.0	1.5	ND	ND	ND
*Defb47*	Defensin beta 47	3.47E-09	7.162	0.2	0.1	31.1	ND	ND	Immune system process, Metabolic process, Response to stimulus

FDR, false-discovery rate; GO, gene ontology; HET, heterozygote; KO, knock-out; ND, not determined; WT, wild-type.

**Table 2:** Top-15 downregulated genes in the testes of PLAG1*-*deficient mice.

Gene symbol	Gene name	FDR	Log fold change	Counts per million			GO terms		
				WT	HET	KO	Molecular function	Cellular component	Biological process
*Dynlt1a*	Dynein light chain Tctex-type 1 A	9.23E-151	−4.525	52.3	51.5	2.3	Binding	Cell part	ND
*Morf4l1*	Mortality factor 4 like 1	8.05E-17	−1.231	128.9	105.7	56.6	Nucleic acid binding transcription factor activity, Binding	Cell part	Metabolic process, Biological regulation
*2700054A10Rik*	RIKEN cDNA 2700054A10 gene	2.81E-16	−1.889	11.1	12.4	3.1	ND	ND	ND
*9430014N10Rik*	RIKEN cDNA 9430014N10 gene	1.72E-15	−6.875	17.1	0.07	0.13	ND	ND	ND
*Prss45*	Protease, serine 45	2.09E-13	−0.961	74.9	78.8	39.7	Catalytic activity, Receptor activity, Binding, Enzyme regulator activity	Extracellular region	Metabolic process, Response to stimulus, Localization. Biological regulation
*Vgll2*	Vestigial like 2 homolog	1.14E-11	−9.220	18.6	0.07	0.03	Nucleic acid binding transcription factor activity, Binding	Cell part	Developmental process
*Sult1e1*	Sulfotransferase family 1E, member 1	1.55E-11	−1.739	53.4	28.7	16.5	Catalytic activity	Cell part	Metabolic process
*Gm13247*	Predicted gene 13247	5.69E-11	−5.049	3.2	0.07	0.1	Nucleic acid binding transcription factor activity, Binding	ND	Metabolic process, Biological regulation
*Olfr183*	Olfactory receptor 183	1.45E-10	−8.618	1.3	0.0	0.0	ND	ND	ND
*4930515B02Rik*	RIKEN cDNA 4930515B02 gene	2.56E-10	−7.418	2.2	0.03	0.0	ND	ND	ND
*Tdgf1*	Teratocarcinoma-derived growth factor 1	2.56E-10	−5.626	0.6	0.8	0.0	Binding	Organelle, Cell part, Extracellular region, Extracellular matrix, Membrane	Cellular process, Developmental process
*Gsta3*	Glutathione S-transferase, alpha 3	1.43E-09	−1.517	63.8	31.7	22.9	Catalytic activity	Cell part, Organelle	Metabolic process, Multicellular organismal process, Development process
*Fnbp1l*	Formin binding protein 1-like	1.75E-09	−2.241	193.1	69.3	42.1	Catalytic activity, Binding, Enzyme regulator activity	Organelle, Intracellular	Metabolic process, Cellular process, Localization, Biological regulation
4930523O13Rik	RIKEN cDNA *4930523O13*	2.24E-09	−3.600	10.6	0.7	0.9	Binding	Organelle, Cell part, Extracellular region, Extracellular matrix, Membrane	Cellular process, Developmental process, Biological regulation
*Amy1*	Amylase 1, salivary	6.60E-09	−1.315	45.1	22.5	18.6	Catalytic activity, Binding	Organelle, Extracellular region, Extracellular matrix	Metabolic process

FDR, false-discovery rate; GO, gene ontology; HET, heterozygote; KO, knock-out; ND, not determined; WT, wild-type.

### Loss of PLAG1 causes defects in spermatogenesis

**Figure 3:**
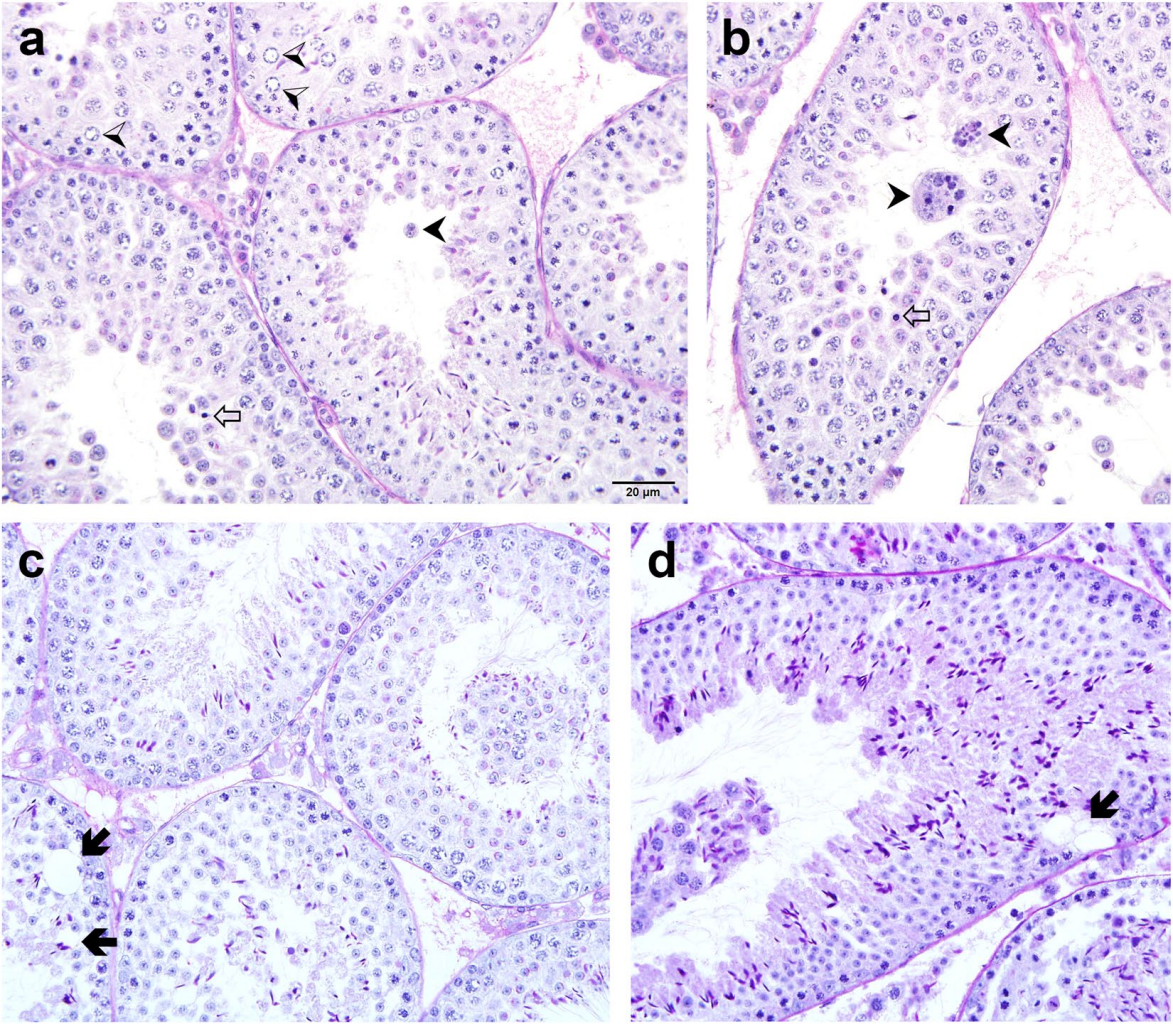
Histology of the testes of *Plag1* knock-out mice. (**a**,**b**) Testis of a 3-week-old male, showing large symplasts (black arrowheads), marginated nuclear chromatin (black-and-white arrowheads) and pyknotic nuclei (open arrows). (**c**,**d**) Testis of a 5-week-old male, showing sloughing of spermatocytes and spermatids, and tubular vacuolation (black arrows). Sections were stained with periodic acid−Schiff and hematoxylin. Magnification as per the scale bar shown in **a**.

The morphological characteristics of the testes were examined by histological analysis of *Plag1* WT and KO mice at various ages between 5 and 36 weeks. WT testes displayed normal histology at all ages analysed. Adult KO males, however, exhibited defects in testicular histology that included the presence of large symplasts, marginated nuclear chromatin, cell death, and tubular and Sertoli cell vacuolation (Fig. 3). The latter is generally considered a sign of recent germ cell loss, which is consistent with the presence of spermatocytes and spermatids in the seminiferous lumen (and caput epididymis). A number of KO animals displayed sloughing of germ cells from the seminiferous epithelium (Fig. 3; Supplementary Fig. S5), in some cases with all cells but spermatogonia detaching. Sloughing of the germ cell epithelium was never observed in WT animals. In a cohort of 11–13-week-old males, a significantly higher fraction of tubules with dying cells and sloughing of the epithelium was found in the KO compared to the WT mice (Supplementary Table S4). TUNEL stain and immunostaining for cleaved Caspase 3 and cleaved Caspase 9 confirmed the identity of cells considered dying as being apoptotic (Supplementary Fig. S6).

### Loss of PLAG1 results in reduced testis and seminal vesicle weight, daily sperm production and sperm motility

**Figure 4:**
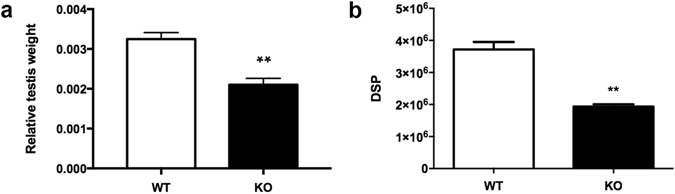
Relative testis weight and daily sperm production of *Plag1* wild-type (WT) and knock-out (KO) mice. (**a**) Testis weight relative to body weight of 11–13-week-old males of WT and KO males (*n* = 4). (**b**) Daily sperm production (DSP) in KO vs WT males at 11–13 weeks of age (*n* = 3). Values shown are means ± SEM. **P < 0.01 (*t*-test).

**Figure 5:**
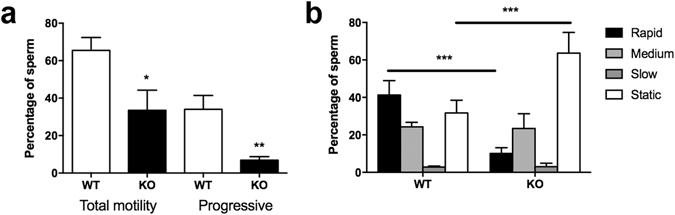
Sperm motility in *Plag1* wild-type (WT) and knock-out (KO) mice. (**a**) Motility of spermatozoa from the cauda epididymis of KO vs WT mice aged 11–13 weeks of age (*n* = 5). (**b**) Velocity distribution of spermatozoa from KO vs WT mice (*n* = 5). Values shown are means ± SEM. *P < 0.05 (*t*-test); **P < 0.01 (*t*-test); ***P < 0.001 (ANOVA).

The weight of testes and seminal vesicles relative to body weight, as well as daily sperm production and sperm motility were compared between age-matched *Plag1* WT and KO mice. Seminal vesicles were found to be disproportionally smaller in 5–6-week-old KO males, weighing on average (with SEM) 0.43 ± 0.06% of body weight in WTs, compared to 0.14 ± 0.02% of body weight in KOs (*t*-test, *P* = 0.0131). Likewise, KO males showed a significant reduction in testis weight relative to body weight compared to WTs at 5–6 weeks of age (WT: 0.31 ± 0.02% vs KO: 0.18 ± 0.02%; *t*-test, *P* = 0.0019), and also at 11–13 weeks of age (*t*-test, *P* = 0.0025) (Fig. 4a). Daily sperm production (DSP) was significantly reduced (−48%) in adult KO males compared to WTs (*t*-test, *P* = 0.0018) (Fig. 4b). Spermatozoa isolated from the cauda epididymis of KO males showed a 49% reduction in motile sperm (*P* = 0.0364) and an 80% reduction in sperm showing progressive motility specifically (*t*-test, *P* = 0.0078), compared to those of age-matched WTs. Furthermore, analysis of velocity revealed a higher fraction of rapid spermatozoa in the WTs compared to the KOs, and a higher fraction of static spermatozoa in the KOs than in the WTs (ANOVA, *P* = 0.0003) (Fig. 5).

## Discussion

Various studies, including several using male mouse models, have revealed a substantial genetic basis of impairment of spermatogenesis, accounting for 10–15% of severe male infertility^10^. However, our understanding of the genetic mechanisms regulating spermatogenesis and sperm function is still poor, as reflected by the large number of male infertility cases of which the underlying molecular cause is unknown. This study aimed to investigate the role of the gene transcription factor PLAG1 in testicular function.

PLAG1 is expressed in the seminiferous tubules of the adult murine testis, in all germ cell types. This is in agreement with an earlier study^14^, in which *Plag1* mRNA was reported to be expressed in spermatogonia, spermatocytes and spermatids, as determined by RNA-sequencing. The expression levels of *Plag1* mRNA decreased as spermatogenesis progressed, having the highest expression in the spermatogonia and preleptotene spermatocytes, and much lower expression in the pachytene spermatocytes and spermatids [supplementary material in ref. 14]. *Plag1* mRNA has also been detected in isolated Sertoli cells by RT-PCR^13^. Like in the germ cells, we found X-gal staining to co-localise with a Sertoli cell marker only occasionally. PLAG1 expression could be subject to posttranscriptional regulation. In human, PLAG1 expression is known to be regulated by various microRNAs, including miR-107, −141, −155, −181a, −181b, and −424, for which conserved binding sites are present in the 3′ untranslated region of the *PLAG1* transcript^15^. Various of these microRNAs are known to be present in the human testis^16^. As the *lacZ* coding sequence in the KO mice is still flanked by the *Plag1* untranslated regions^13^, translation of the *Plag1* mRNA may be inhibited by miRNAs, resulting in limited protein expression. However, it is not clear what causes PLAG1 to be expressed in only some cells and not others of the same type. PLAG1 deficiency in itself could affect the expression sites of PLAG1, but we found the same expression pattern in heterozygous mice. Further analysis of the testicular PLAG1 expression pattern using immunohistochemistry would be beneficial to confirm the X-gal staining pattern reported here, but so far, we have not found antibodies that resulted in convincing, specific staining of murine PLAG1.

Transcriptome analysis of the testes revealed four notable effects of PLAG1 deficiency: (1) an overall downregulation of genes involved in spermatogenesis; (2) a downregulation of key genes involved in steroidogenesis; (3) an upregulation of several immunity-related genes; and (4) an induction of testicular expression of epididymis-specific genes.

Several genes that were downregulated in the KO testis are known to be involved in spermatogenesis, for example deleted in azoospermia-like (*Dazl*), stimulated by retinoic acid gene 8 (*Stra8*) and Piwi-like protein 1 (*Piwil1*). *Dazl* is required for meiosis, and DAZL-deficient mice lack gametes, because the germ cells do not progress through meiosis^17^. Recent studies have shown that DAZL is involved in regulating pluripotency, differentiation and apoptosis in developing germ cells^18, 19^, and the association of DAZL with 10−15% of human male infertility cases has been reviewed^20, 21^. Likewise, *Stra8* is required for progression of the meiotic cell cycle^22^. It is expressed in meiotic and postmeiotic germ cells^23^, and STRA8*-*deficient spermatocytes initiate but fail to complete meiosis and undergo premature chromosome condensation^22^. *Piwil1* is known to be expressed in spermatocytes and spermatids, with PIWIL1-deficient mice exhibiting spermatogenic arrest^24–26^.

Daily sperm production was significantly lower in the KO mice compared to the WT and, in contrast to an earlier report^13^, we found the testes of the *Plag1* KO males to be disproportionally smaller than those of WTs. The reduction in DSP is very likely to contribute in a major way to the fertility issues in the KO males. Additional spermatogenic aberrations, such as a higher number of dying cells and sloughing of the germinal epithelium, were observed in adult males, and these may be linked to the changes in the expression of genes involved in spermatogenesis observed in the transcriptome study. While all stages of germ cell development were typically present, some *Plag1* KO males exhibited sloughing of spermatids and spermatocytes, and in some cases, almost complete detachment of the germinal layer. This defect was reminiscent of the sloughing reported in the seminiferous tubules of rats treated with the microtubule-disrupting chemicals colchicine and vinblastine^27^, where germ cells and apical parts of Sertoli cells were sloughed together and remained in close association in the lumen of the seminiferous tubule. In addition, germ cell death and formation of often large symplasts was evident in the seminiferous tubules of KO mice, the latter particularly in younger mice. Symplasts originate when the intercellular bridges between round spermatids collapse and form a multinucleated cell that displays arrested development. These symplasts eventually undergo degeneration and are phagocytised by Sertoli cells and intratubular macrophages^28^.

Importantly, PLAG1-deficient mice not only showed greatly reduced sperm output, their sperm also had significantly decreased motility compared to WTs. Such asthenozoospermia is a clinical sign of infertility with multiple potential causes whose identification requires assessment of the various mechanisms and signalling pathways that regulate sperm motility, as well as the gross and ultrastructural morphology of sperm, in particular the flagellum^29^. It remains unknown how PLAG1 affects sperm motility, but the reduced sperm motility seen in KO males in combination with the reduced DSP mentioned above, is likely to be a crucial contributing factor in the subfertility observed in male PLAG1-deficient mice.

A few genes encoding key enzymes of the steroidogenic pathway were differentially expressed. *Sultle1* and *Hsd17b3* were both downregulated. *Hsd17b3* encodes the enzyme 17β-hydroxysteroid dehydrogenase type 3, which catalyses the conversion of androstenedione to testosterone. *Sult1e1* protects the Leydig cells from estrogen-induced aberrations in cholesterol uptake and steroidogenesis by catalysing sulfoconjugation and inactivation of oestrogens^30^; loss of *Sult1e1* results in decreased production of testosterone^31^. These observations indicate lower testosterone levels in PLAG1-deficient mice. We have previously suggested that *Plag1* may be required for normal androgen balance^12^, because *Plag1* KO mice have disproportionally smaller seminal vesicles and ventral prostate than WT mice, despite the absence of *Plag1* expression in these organs in WT animals^13^. The disproportionally small size of the seminal vesicles was confirmed in the present study. In addition, most of the Leydig cell markers were downregulated, except *9230104L09Rik* (cystatin E2 gene) and *Slc38a1*, which were upregulated. Cystatin E2 can be suppressed by androgen administration to castrated mice^32^. Accordingly, upregulation of cystatin E2 in the *Plag1* KO mice may again point to a decrease of testosterone. The effects of PLAG1 deficiency on androgen production must be indirect, since we found no PLAG1 expression in Leydig cells.

GO analysis of the differentially expressed genes showed that ~10% of the upregulated genes in the KO testes are involved in immune system processes (GO:0002376). Steroidogenesis, spermatogenesis and the immune system are linked via a complex network of interactions. An intricate balance of these testicular functions is required such that immune suppression is maintained to protect the germ cells from auto-attack and at the same time preserve the ability to illicit an immune response during infection^33, 34^. Some genes involved in inflammation were upregulated, such as interleukin 1 receptor antagonist (*Il1rn*) and several receptors, including macrophage mannose receptor 1 (*Mrc1*), leukocyte immunoglobulin-like receptor subfamily B member 4 (*Lilrb4*), killer cell lectin-like receptor subfamily B member 1 C (*Klrb1c*), colony-stimulating factor 1 receptor (*Csf1r*), and tumor necrosis factor receptor superfamily member 25 (*Tnfrsf25*). The upregulated adhesion G protein-coupled receptor E1 (*Adgre1*) gene is responsible for the production of CD8 + lymphocytes^35^, and the presence of these cells in the testis is associated with subfertility or infertility, at least in humans^36^. In addition, several β-defensin genes were upregulated in the KO testes, with *Defb25* and *Defb47* in the top 15 of upregulated genes. β-Defensins have been shown to be preferentially expressed in the male reproductive tract, more in the epididymis than in the testis, where they have antimicrobial functions to protect the sperm from pathogens^37^. Lastly, the Cd68 antigen gene was upregulated in the KO mice. This gene is expressed by a subpopulation of testicular macrophages that have the ability to secrete pro-inflammatory cytokines in rats^36^. Further investigation of the immune status of the KO mice is important in order to deduce whether the potential upregulation of immune processes is having an additional impact on the fertility of the PLAG1-deficient male mice.

Intriguingly, several of the upregulated genes in the testis of KO mice were genes that are normally only or mostly found in the epididymis. The most significantly upregulated gene in the testes of KO mice was lipocalin 9 (*Lcn9*). Three other lipocalins −*Lcn6*, *Lcn8*, and *Lcn10*− were also among the upregulated genes in the KO testes. Lipocalins are transport proteins of small hydrophobic ligands such as steroids and retinoids; they are normally exclusively expressed in the epididymis and have been shown to be absent in the normal testes^38^. This was confirmed by the testicular transcriptome of the WT mice. The third most upregulated gene in the KO testes was *Teddm1*, encoding transmembrane epididymal protein 1 A. Although this gene’s function has not been determined, it is also specifically expressed in the epididymis^39^. Similarly, the transmembrane epididymal family member 2 (*Teddm2*) gene with unknown function was among the top-15 upregulated genes. Additional epididymal genes that were expressed in the KO testes are glutathione peroxidase 5 (*Gpx5*) and cystatin 11 (*Cst11*). GPX5 protects sperm against oxidative damage^40^. CST11 is located on the surface of sperm cells where it functions as an antimicrobial protein, protecting the sperm from pathogens in the male reproductive tract, and as a defence when exposed to the female reproductive tract^41^. The upregulated genes A disintegrin and metalloprotease domain 7 (*Adam7*) and *Adam28* are normally predominantly expressed in the epididymis where their gene products interact with sperm to ensure motility and fertilisation capacity^42, 43^. They are regulated by androgens and/or other testicular factors and are expressed in a region-specific manner within the epididymis^44, 45^. Lastly, the adhesion G protein-coupled receptor G2 (*Adgrg2*) gene was highly upregulated in the testes of PLAG1*-*deficient mice. This gene is normally expressed in the efferent ductiles and the initial section of the epididymis. Interestingly, epididymis-specific mRNAs that were downregulated by disruption of *Adgrg2* in an earlier study^45^ include *Defb42*, *Lcn8, Lcn9*, *Adam28* and *Slc1a1*, which were all significantly upregulated in the testes of PLAG1*-*deficient mice. The abnormal expression of these genes in the testes of the *Plag1* KO mice shows that in the absence of *Plag1*, epididymis-specific genes are being switched on, and that under normal conditions, PLAG1 directly or indirectly suppresses the testicular expression of epididymis-specific genes. Whether or not the proteins encoded by these epididymal genes have a functional role in the KO testis is not known, but given that a number of them play a role in the protection of sperm against pathogens, their appearance in the KO testis may be linked with the upregulation of genes involved in immune processes.

In summary, PLAG1 is expressed in germ cells and Sertoli cells in the seminiferous tubules of adult mice. Based on GO analysis of differentially expressed genes in the testes of PLAG1-deficient mice versus WT mice, there is an overall downregulation of genes involved in spermatogenesis and upregulation of genes involved in immune responses and epididymal processes. In addition, there are several indications that androgen synthesis is affected in *Plag1* KO mice. Daily sperm production was greatly reduced in *Plag1* KO mice. Finally, sperm motility was found to be significantly affected in *Plag1* KO males. While other potential contributing factors, such as alterations in sexual behaviour and mating frequency, remain to be investigated, our results demonstrate that PLAG1 is important in testicular function and sperm motility, calling for the contribution of PLAG1 mutations or polymorphisms to male infertility in humans to be examined.

## Materials and methods

### Animals

All animal procedures were approved by the Animal Ethics Committee of La Trobe University (AEC13-08) and the La Trobe Institutional Biosafety Committee (GMSC13-03), and were carried out in accordance with the Australian Code of Practice for the Care and Use of Animals for Scientific Purposes (7th edition, 2004) of the National Health and Medical Research Council. The generation of *Plag1* KO mice and their genotyping have been described previously^13^.

### X-gal staining

KO, HET and WT male mice of either 5 weeks old or >2 months old were euthanized by CO_2_ asphyxiation, and testes were dissected and immediately placed in 4% (w/v) paraformaldehyde in phosphate-buffered saline (PBS; pH 7.6) for 2 h at 4 °C. The tissues were cryoprotected in 10% (w/v) sucrose in PBS for 2 h followed by 30% sucrose in PBS overnight. The tissues were then embedded in 10% (w/v) gelatin and 30% sucrose in PBS, and stored at −80 °C until cryosectioning. Frozen testes were sliced into 5-μm (5 weeks old) or 12-μm (>2 months old) cryosections and mounted onto glass slides. The sections were thawed at room temperature for 30 min and then incubated in staining solution containing 0.5 mg/ml X-gal (5-bromo-4-chloro-3-indoyl-β-D-galactopyranoside; PanReac Applichem, Darmstadt, Germany), 5 mM K_3_Fe(CN)_6_, 5 mM K_4_Fe(CN)_6_, 2 mM MgCl_2_, 0.01% (w/v) sodium deoxycholate, 50 mM EGTA, and 0.02% (v/v) IGEPAL CA-630 (Sigma-Aldrich, St. Louis, MO, USA) in PBS (pH 7.4) in the dark at 37 °C overnight. The samples were then fixed in 4% paraformaldehyde or a mixture of 2% paraformaldehyde and 0.2% (v/v) glutaraldehyde in PBS for 10 min, and rinsed in PBS. Some sections were counterstained using Nuclear Fast Red (Sigma-Aldrich) prior to mounting with dibutylphthalate polystyrene xylene, and then imaged using an Olympus BX41 microscope with DP25 camera (Olympus Scientific Solutions Americas Inc, Waltham, MA, USA).

### Immunohistochemistry

Five-μm cryosections of testes from 5-week-old animals were washed three times 5 min in PBS following X-gal staining and then underwent antigen retrieval (30 min in boiling 10 mM trisodium citrate) and pre-treatment for 15 min in 10% (v/v) methanol and 3% (v/v) H_2_O_2_ in PBS. Slides were incubated for 1 h in 5% BSA in PBS at 4 °C and then washed three times 5 min in PBS before being incubated overnight at 4 °C with goat polyclonal anti-mouse GATA4 (Santa Cruz Biotechnology, Dallas, TX, USA; catalogue number sc-1237; RRID: AB_2108747) diluted 1:200 in 1% BSA in PBS. Next, sections were washed three times 5 min in PBS, incubated for 1 h at room temperature with biotinylated mouse anti-goat IgG (Santa Cruz Biotechnology; catalogue number sc-2489; RRID: AB_628488) diluted 1:100 1% BSA in PBS, and again washed three times 5 min in PBS before incubation with streptavidin−horseradish peroxidase (Dako) at a dilution of 1:400 in 1% BSA in PBS for 1 h at room temperature. The sections were washed three times 5 min in PBS, and signal visualized with 1 mg/ml diaminobenzidine and 0.01% H_2_O_2_ in PBS. Sections were washed three times 5 min with PBS and coverslipped using Glycergel.

### RNA isolation, library construction and RNA-seq

Testes from nine mice (three KO, three HET and three WT animals), aged 5 weeks, were collected and stored in RNA*later* at −80 °C. Testes were homogenized and total RNA was extracted using TRIzol Reagent (Invitrogen, Carlsbad, CA, USA) according to the manufacturer’s guidelines. The extracted RNA was treated with DNase I using the TURBO DNA-*free* kit (Invitrogen) to remove genomic DNA and purified using the Zymo RNA Clean & Concentrator-5 (Zymo Research Corporation, Irvine, CA, USA). RNA quality was determined using the Agilent RNA 6000 Nano kit on an Agilent 2100 Bioanalyzer (Agilent Technologies, Santa Clara, CA, USA); RNA Integrity Number (RIN) values ranged from 7 to 9. The RNA concentration was measured using the Qubit RNA HS Assay kit on the Qubit 3.0 Fluorometer (Invitrogen) and then an equal starting quantity of 500 ng of total RNA for each of the samples underwent poly(A) selection.

Nine sequencing libraries were constructed with the TruSeq Stranded mRNA LT - Set A kit (Illumina, San Diego, CA, USA) as per the manufacturer’s instructions. Library quality was determined using an Agilent DNA 1000 kit on the Agilent 2100 Bioanalyzer, and the concentration was determined using the Qubit dsDNA HS Assay kit on the Qubit 3.0 Fluorometer. The average fragment size for each library was ~250 bp. In the final step prior to sequencing, the nine individually indexed libraries were pooled in equimolar quantities, and sequenced using 150-bp paired-end sequencing on a single lane of an Illumina NextSeq 500, generating approximately 50 million 150-bp paired-end reads per library. The raw data have been submitted to the NCBI Sequence Read Archive, accession numbers SAMN06165292–SAMN06165294, SAMN06165301–SAMN06165303 and SAMN06165310–SAMN06165312.

### RNA-seq data analysis

The raw sequencing data were initially assessed using FastQC (http://www.bioinformatics.babraham.ac.uk/projects/fastqc/). The paired-end reads were merged to a single read using PEAR^46^, with 97% of reads properly overlapping. The resulting high-quality single reads were mapped to the mouse genome (version mm10) using TopHat2 version 2.1.0^47^ with Bowtie2 version 2.2.6^48^. Expression counts were determined for each gene using htseq-count and the standard mm10 gene annotations. Standard differential expression analysis with an FDR cut-off of 0.05 was performed using the edgeR programme as implemented in Degust (http://www.vicbioinformatics.com/degust).

GOrilla^49, 50^ and PANTHER^51, 52^ GO analysis tools were used to identify biological pathways that were enriched and to categorise genes according to GO terms in the different data sets. These programmes automatically obtain the GO annotations and generate functional annotations that are overrepresented in the input list of genes, in order to identify relationships between the genes of interest and to uncover common processes and pathways.

### Quantitative PCR

Approximately 2 µg of DNase I-treated testis total RNA (*n* = 3 for each of WT and KO) was reverse transcribed to cDNA in a volume of 40 µl containing M-MuLV reverse transcriptase reaction buffer (50 mM Tris-HCl, 75 mM KCl, 3 mM MgCl_2_, 10 mM dithiothreitol, pH 8.3; New England BioLabs), 540 ng oligo(dT)_18_, 0.5 mM of each dNTP, 80 U RNasin ribonuclease inhibitor (Promega, Madison, WI, USA) and 100 U MultiScribe reverse transcriptase (ThermoFisher Scientific). The mixture was incubated for 10 min at 25 °C, 2 h at 42 °C and 5 min at 85 °C, after which the cDNA samples were diluted 1:5 in sterile, RNase-free water. Quantitative PCR was used to determine expression levels of *Hsd17b3, Srd5a2, Lcn9, Defb25*, and of the reference gene *Actb*, whose expression levels were not altered by PLAG1 deficiency according to the transcriptome study. Primer sequences are listed in Supplementary Table S5. Each qPCR amplification reaction mixture had a final volume of 20 µl and contained 5 μl of diluted cDNA, 300 nM of forward and reverse primer, and 1× Fast SYBR Green Master Mix (Applied Biosystems). Quantitative PCR was performed using the 7500 Fast Real-Time PCR System (Applied Biosystems). After 20 s at 95 °C, samples went through 40 cycles of 3 s at 95 °C and 30 s at 60 °C. A dissociation protocol followed the amplification programme to detect non-specific amplification. Each individual sample was measured in triplicate and a no-template and no-reverse-transcription control were added as negative controls. The starting concentration of each sample, expressed in arbitrary fluorescence units, was calculated based on the mean PCR efficiency per amplicon and the quantification cycle value per sample using LinRegPCR software^53^. These values were then normalised against the starting concentrations of *Actb* for each sample. Unpaired *t*-tests were performed in GraphPad Prism 7 (GraphPad Software, La Jolla, CA, USA) to compare the average normalised expression levels in KOs and WTs. Means were considered significantly different if *P* < 0.05.

### Testis and seminal vesicle weight

Testis weight relative to body weight was determined in four KO and four WT males aged between 11 and 13 weeks, and in eight KO and three WT males aged between 5 and 6 weeks. An additional three KO and three WT males aged 5–6 weeks were used to determine the weight of the seminal vesicles relative to body weight. Unpaired *t*-tests were performed in GraphPad Prism 7. Means were considered significantly different if *P* < 0.05.

### Testis histology

Male KO mice aged 3 (*n* = 1), 5 (*n* = 1), 11–13 (*n* = 5), 18–23 (*n* = 4), 29 (*n* = 1), 36 (*n* = 1), and 54 weeks (*n* = 1) and WT mice aged 5 (*n* = 1), 11–13 (*n* = 5), 18−23 (*n* = 4), 29 (*n* = 1), and 36 (*n* = 1) weeks were used for histological examination of the testes. After piercing the tunica albuginea, the testes were placed in Bouin’s fixative (Amber Scientific, Midvale, WA, Australia) for 5 h at room temperature and transferred to 70% (v/v) ethanol in water until ready for further processing. Tissues were immersed for 2 h in each of the following: 70% ethanol, 90% ethanol, 100% ethanol, 100% ethanol, 50:50 ethanol:xylene, 100% xylene, paraffin wax (60 °C), paraffin wax (60 °C), and finally embedded in paraffin wax. This was followed by sectioning at a thickness of 5 μm. The sections were stained using periodic acid−Schiff (PAS) and counterstained using hematoxylin as described by Ahmed and de Rooji^54^. The sections were de-waxed and rinsed, followed by staining in 0.55% (v/v) periodic acid (Amber Scientific) in distilled water for 10 min, and rinsed under running tap water for 5 min. They were then stained with Schiff’s reagent for 20 min and rinsed under warm running water for 10 min. After the PAS stain, the sections were counterstained with Mayer’s haematoxylin (Amber Scientific) for 1 min and washed in running tap water for 5 min before being immersed in Scott’s tap water substitute (Amber Scientific) for 60 s. After washing in running tap water for 5 min, sections were dehydrated in ascending grades of ethanol: 70% ethanol, 100% ethanol, 100% ethanol, 100% ethanol, cleared in 100% xylene, and mounted with dibutylphthalate polystyrene xylene. The histology of the tissues was compared between age-matched KO and WT mice where possible.

### Daily sperm production

Testes from 11–13-week-old KO and WT males (*n* = 3 per genotype) were dissected, weighed and stored at −20 °C until use. Testicular DSP was determined as described by Cotton *et al*.^55^. Each testis was homogenised in 400 μl DSP buffer (0.9% (w/v) M NaCl, 0.01% (w/v) NaN_3_ and 0.05% (v/v) Triton X-100 in water). Each sample was further diluted 1:2 in DSP buffer. The cells were counted for each testis in a haemocytometer and counts for six chambers were averaged. Daily sperm production was determined by dividing the number of elongated spermatids per testis by 4.84, which is the number of days that developing spermatids spend in stage 14–16 during spermatogenesis in the mouse^56^. Statistical analyses were performed in GraphPad Prism 7. Unpaired *t*-tests were used for all comparisons. Means were considered significantly different if *P* < 0.05.

### Sperm motility

Sperm were back-flushed from the cauda epididymis of 11–13-week-old males (*n* = 5 of each WT and KO). Each epididymis was dissected and placed in mineral oil pre-warmed at 37 °C. The sperm were gently released into 2 ml of equilibrated Modified Tyrode’s 6 (MT6) medium (0.73% NaCl, 0.02% (w/v) KCl, 0.01% (w/v) MgCl_2_.6H_2_O, 0.005% (w/v) NaH_2_PO_4_.H_2_O, 0.1% (w/v) D-Glucose, 0.21% (w/v) NaHCO_3_, 0.001% (w/v) Phenol Red, 0.025% (w/v) CaCl_2_.2H_2_O and 0.4% (w/v) BSA in water), which was incubated for 90 min in an incubator at 37 °C with 5% CO_2_. For some of the KO epididymides that were too small to back-flush, small nicks were carefully made into the cauda to release the sperm. After a 90-min incubation, 10 µl of each sample was added onto each chamber of a pre-warmed 2X Cel Slide (Hamilton Thorne, Beverly, MA, USA). The slide was placed onto a MiniTherm Slide warmer (Hamilton Thorne) and visualised under the 4× objective of the Olympus CX41 microscope. Motility was analysed using the MouseTraxx Sperm Analysis System (Hamilton Thorne) to quantify the total and progressive motility, as described by Gibbs *et al*.^57^. We aimed to score a minimum of 1000 sperm in duplicate per animal, but this was not always possible for the KO males, due to low sperm numbers. Sperm from KO males were compared to age-matched WT males using unpaired *t*-tests and a two-way ANOVA was used to compare velocity distribution. Means were considered significantly different if *P* < 0.05.

## Electronic supplementary material

Supplementary Information
