# Supplementary material for: PLAG1 deficiency impairs spermatogenesis and sperm motility in mice
Source: Sci Rep. 2017 Jul 13;7:5317. doi: 10.1038/s41598-017-05676-4 (PMC5509656; doi:10.1038/s41598-017-05676-4)
Supplement: Supplementary file 1 — Supplementary Information [file 41598_2017_5676_MOESM1_ESM.pdf]

Supplementary Information

PLAG1 deficiency impairs spermatogenesis and sperm motility in mice

Almas R. Juma, Sylvia V.H. Grommen, Moira K. O'Bryan, Anne E. O'Connor, D. Jo Merriner, Nathan E. Hall, Stephen R. Doyle, Pauliina E. Damdimopoulou, Daniel Barriga, Adam H. Hart, Wim J.M. Van de Ven, Bert De Groef

Supplementary Table S1. 'Biological process' gene ontology terms enriched among differentially expressed genes in the testes of PLAG1-deficient mice, from a total of 18,525 expressed genes.

GO term	Description of biological process	Number of genes
**Upregulated genes**		
GO:0002376	Immune system process	13
GO:0006955	Immune response	77
GO:0006952	Defense response	84
GO:0098542	Defense response to other organism	45
GO:0022610	Biological adhesion	90
GO:0045087	Innate immune response	46
GO:0007155	Cell adhesion	88
GO:0043207	Response to external biotic stimulus	63
GO:0051707	Response to other organism	49
GO:0042742	Defense response to bacterium	32
GO:0045765	Regulation of angiogenesis	39
GO:0009607	Response to biotic stimulus	63
GO:0009617	Response to bacterium	33
GO:1901342	Regulation of vasculature development	39
GO:0050896	Response to stimulus	223
GO:0048002	Antigen processing and presentation of peptide antigen	13
GO:0070208	Protein heterotrimerization	6
GO:0051704	Multi-organism process	61
GO:0009605	Response to external stimulus	94
GO:0034097	Response to cytokine	36
GO:0045766	Positive regulation of angiogenesis	25
GO:0071310	Cellular response to organic substance	42
GO:0050776	Regulation of immune response	59
GO:0002675	Positive regulation of acute inflammatory response	6
GO:0070206	Protein trimerization	7
GO:0002252	Immune effector process	36
GO:0030155	Regulation of cell adhesion	64
GO:0030199	Collagen fibril organization	10
GO:0010033	Response to organic substance	112
GO:0090050	Positive regulation of cell migration involved in sprouting angiogenesis	4
**Downregulated genes**		
GO:0046688	Response to copper ion	4
GO:1902340	Negative regulation of chromosome condensation	2
GO:0009566	Fertilization	16
GO:0007338	Single fertilization	9
GO:0022414	Reproductive process	81
GO:0006641	Triglyceride metabolic process	5
GO:0044702	Single organism reproductive process	76
GO:0022412	Cellular process involved in reproduction in multicellular organism	30
GO:0007126	Meiotic nuclear division	9

Supplementary Table S2. Differentially expressed genes categorised under the GO term 'Reproduction processes' (GO: 0000003) in *Plag1* knock-out versus wild-type mice.

Gene symbol	Gene name	FDR	Log fold change	Counts per million		
				WT	HET	KO
**Upregulated genes**						
Ovch2	Ovochymase-2	8.46E15	9.76	0.0	0.03	33.6
Adam28	A disintegrin and metalloproteinase domain-containing protein 28	1.84E-14	10.46	0.03	0.03	45.4
Gpr64 (Adgrg2)	G protein-coupled receptor 64	3.90E-08	5.21	0.9	2.5	35.7
Ccnd2	G1/S-specific cyclin-D2	4.14E-05	1.22	11.97	20.03	28.57
Upk1b	Uroplakin-1b	5.77E-03	1.97	0.7	0.87	2.73
Adam7	A disintegrin and metalloproteinase domain-containing protein 7	0.02	2.85	79.8	10.4	588.6
Emr1 (Adgre1)	EGF-like module-containing mucin-like hormone receptor-like 1	0.02	1.12	3.2	3.77	7.06
1700080O16Rik	1700080O16Rik protein	0.04	0.28	32.76	29.37	41.13
Crip1	Cysteine-rich protein 1	0.04	2.79	5.26	15.4	37.76
Hoxb9	Homeobox protein Hox-B9	0.04	1.76	0.63	1.13	2.26
**Downregulated genes**						
Adam26a (*Testase 3*)	A disintegrin and metalloproteinase domain-containing protein 26A	3.08E-06	-0.73	76.8	50.7	47.53
Tlx1 (*Hox11*)	T-cell leukemia homeobox protein 1	2.87E-04	-4.01	1.6	0.1	0.1
Adam25 (*Testase 2*)	A disintegrin and metalloproteinase domain-containing protein 25	1.38E-03	-0.49	71.4	52.6	52.4
Hoxa4	Homeobox protein Hox-A4	0.01	-0.79	90.17	89.93	53.67
Adam21	A disintegrin and metalloproteinase domain-containing protein 21	0.01	-0.63	57.83	48.1	38.6
Piwil1	Piwi-like protein 1	0.02	-0.24	469.6	586.67	411.1
Dazl	Deleted in azoospermia-like	0.03	-0.4	165.8	128.07	129.5
Stard6	StAR-related lipid transfer protein 6	0.03	-0.2	81.77	60.63	73.4

FDR, false-discovery rate; HET, heterozygote; KO, knock-out; WT, wild-type

Supplementary Table S3. Expression levels of selected genes in the testes of *Plag1* wild-type, heterozygous and knock-out mice. An FDR value < 0.05 indicates significant differential expression.

Gene symbol	Gene name	FDR	Log fold change	Counts per million		
				WT	HET	KO
**Sertoli cell markers**						
Ccnd2	Cyclin D2	4.14E-05	1.22	11.9	20.0	28.6
Kitl	Kit ligand	2.33E-03	-0.41	17.8	9.9	13.8
Espn	Espin	0.01	0.56	77.8	98.9	118.2
Mrc1	Mannose receptor, C type 1	0.02	0.73	32.3	38.3	55.4
Frzb	Frizzled-related protein	0.02	1.00	16.0	40.0	32.9
Slc7a1	Solute carrier family 7 (cationic amino acid transporter, y+ system), member 1	0.02	0.37	38.2	56.8	50.8
Daam2	Dishevelled associated activator of morphogenesis 2	0.03	0.44	106.2	119.4	148.6
Shbg	Sex hormone binding globulin	0.04	0.84	8.4	15.5	15.4
Mcf2	Mcf.2 transforming sequence	0.05	0.76	8.03	9.8	14.03
**Leydig cell markers**						
Amy1	Amylase 1, salivary	6.60E-09	-1.32	45.1	22.5	18.6
Fetub	Fetuin beta	1.03E-08	-2.44	3.9	0.7	0.8
Apoc1	Apolipoprotein C-I	3.51E-06	-1.48	27.5	19	10.2
Itih2	Inter-alpha trypsin inhibitor, heavy chain 2	4.13E-05	-1.13	55.6	25	26.0
Lrg1	Leucine-rich alpha-2-glycoprotein 1	8.64E-04	-1.10	9.7	15.2	4.7
Insl3	Insulin-like 3	1.29E-03	-0.79	102.1	87.1	60.8
9230104L09 Rik	RIKEN cDNA 9230104L09 (mCST E2)	3.41E-03	1.48	9.4	4.6	27.3
Klk1b22	Kallikrein 1-related peptidase b22	0.01	-1.77	2.9	0.5	0.9
Slc38a1	Solute carrier family 38, member 1	0.02	1.00	9.9	16.4	20.4
**Germ cell markers**						
Stra8	Stimulated by retinoic acid gene 8	0.03	-0.46	11.8	15.8	8.8
Dazl	Deleted in azoospermia-like	0.03	-0.40	165.8	128.1	129.5
Zpbp	Zona pellucida binding protein	0.25	-0.24	322.5	271.9	280.9
Klhl10	Kelch-like 10	0.36	-0.18	508.5	439.3	461.7
Kit	Kit oncogene	0.59	-0.06	35.6	43.6	35.1
Gapdhs	Glyceraldehyde-3-phosphate dehydrogenase, spermatogenic	0.67	0.02	1206.4	1134.1	1259.4
Tex11	Testis expressed gene 11	0.76	-0.28	20.0	19.2	17.0
Zbtb16	Zinc finger and BTB domain containing 16	0.95	0.03	4.9	5.8	5.2
**Steroidogenic enzymes**						
Sult1e1	Sulfotransferase family 1E, member 1	1.55E-11	-1.74	53.4	28.7	16.5
Srd5a2	Steroid 5 alpha-reductase 2	4.31E-03	2.5	0.4	0.4	2.3
Hsd17b3	Hydroxysteroid (17-beta) dehydrogenase 3	7.35E-03	-0.70	68.4	70.2	43.3
**Hormone receptors**						
Lhcgr (Lhr)	Luteinizing hormone/ choriogonadotropin receptor	0.99	0.08	16.6	17.8	18.0
Fshr	Follicle stimulating hormone receptor	0.93	0.32	3.6	4.2	4.7

FDR, false-discovery rate; HET, heterozygote; KO, knock-out; WT, wild-type

Supplementary Table S4. Occurrence of aberrations in the seminiferous tubules of *Plag1* wild-type and knock-out mice of 11–13 weeks old.

	Wild-type	Knock-out
Symplasts	0.02 ± 0.01	0.02 ± 0.01
Marginated chromatin	0.05 ± 0.02	0.12 ± 0.06
Vacuolation	0.05 ± 0.02	0.06 ± 0.01
Pyknotic nuclei / necrotic cells	0.29 ± 0.05	0.54 ± 0.06**
Sloughing of epithelium	0.00 ± 0.00	0.22 ± 0.06**

Ten to 18 random intact tubules were observed in a randomly selected section of each testis of each animal (n=5 per genotype). The fraction of observed tubules showing one or more symplasts, spermatocytes with marginated chromatin, vacuolation, pyknotic nuclei and/or necrotic or apoptotic cells, and sloughing of the germ cell epithelium was counted. Values are means ± SEM. **P<0.01 (t-test)

Supplementary Table S5. Primers used for quantitative PCR analysis.

Gene	Gene ID	Primer sequences (5'→3')
Hsd17b3	15487	Forward: ATTTTACCAGAGAAGACATCT<br>Reverse: GGGGTCAGCACCTGAATAATG
Srd5a2	94224	Forward: TGGGTCTCTTCTCCGCACAT<br>Reverse: GCCTCTGGTGAGCAATGAGTAA
Lcn9	77704	Forward: CGATTCCATAGCCTCAGATAAC<br>Reverse: GCTGTAGTCGGTCTCCAA
Defb25	654459	Forward: AGTCATGTACCACCAGGAAGCA<br>Reverse: GAAGGCTTGAAAGAATAGGACAGG
Actb	11461	Forward: GGCTGTATTCCCCTCCATCG<br>Reverse: CCAGTTGGTAACAATGCCATGT

a

b

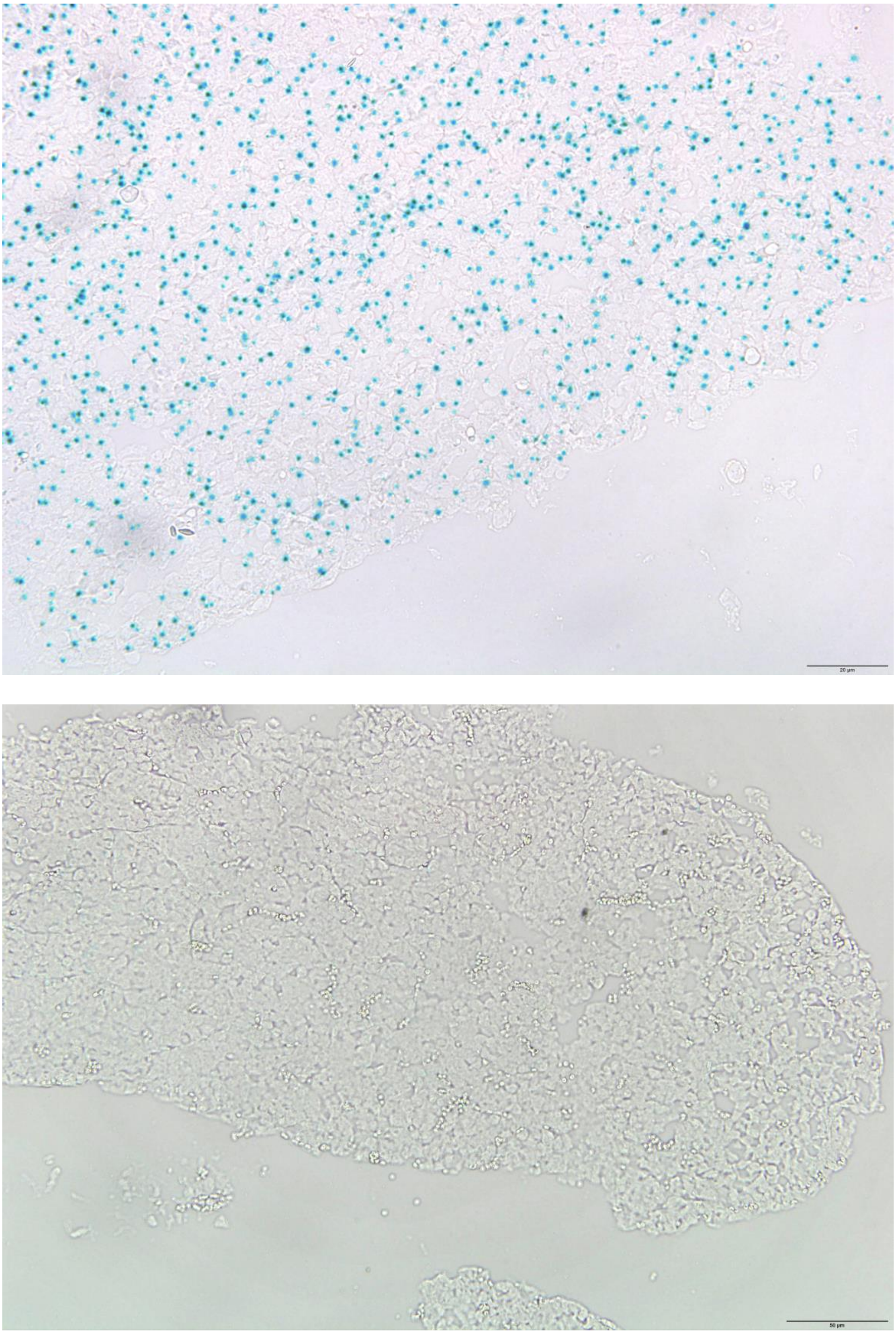

Supplementary Figure S1. Presence of PLAG1 in mouse adenohypophysis, as determined by X-gal staining of cryosections of pituitary glands from *Plag1* knock-out mice in which the coding sequence of *Plag1* was replaced with that of *lacZ*. (**a**) X-gal-positive cells (blue) in the adenohypophysis of a knock-out animal. (**b**) Absence of X-gal signal in the adenohypophysis of a wild-type animal (negative control). Section thickness was 7 μm. Scale bars represent 50 μm.

a

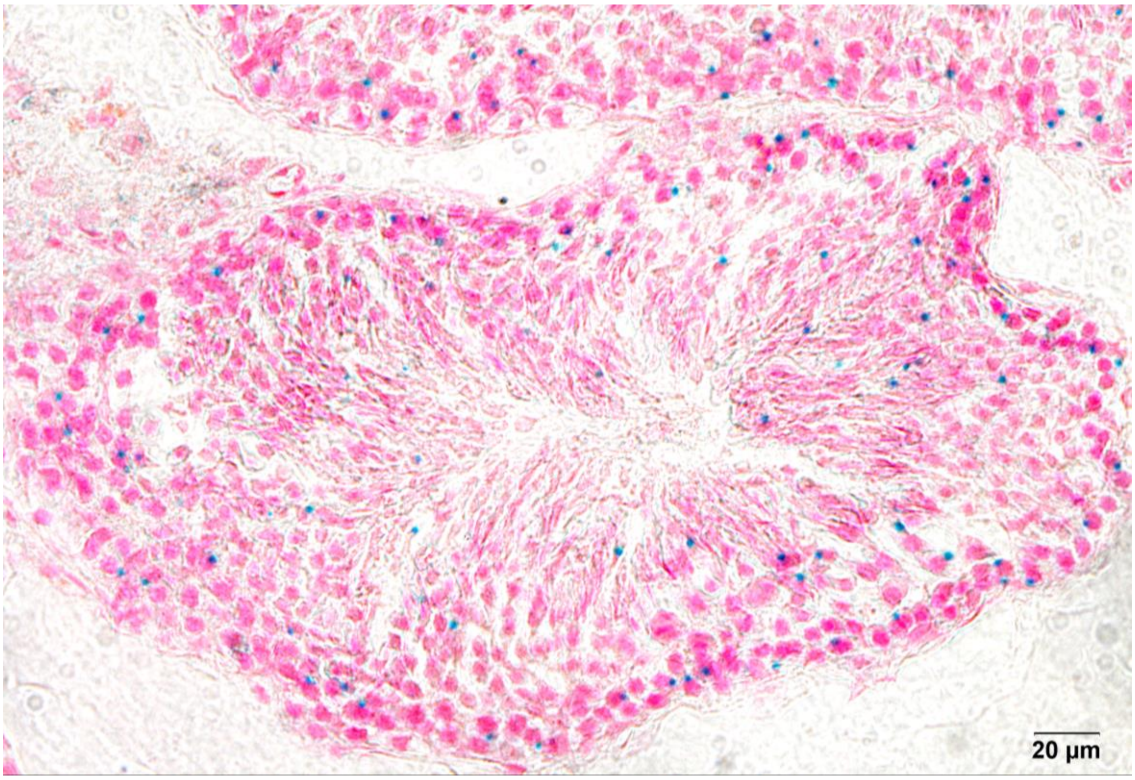

b

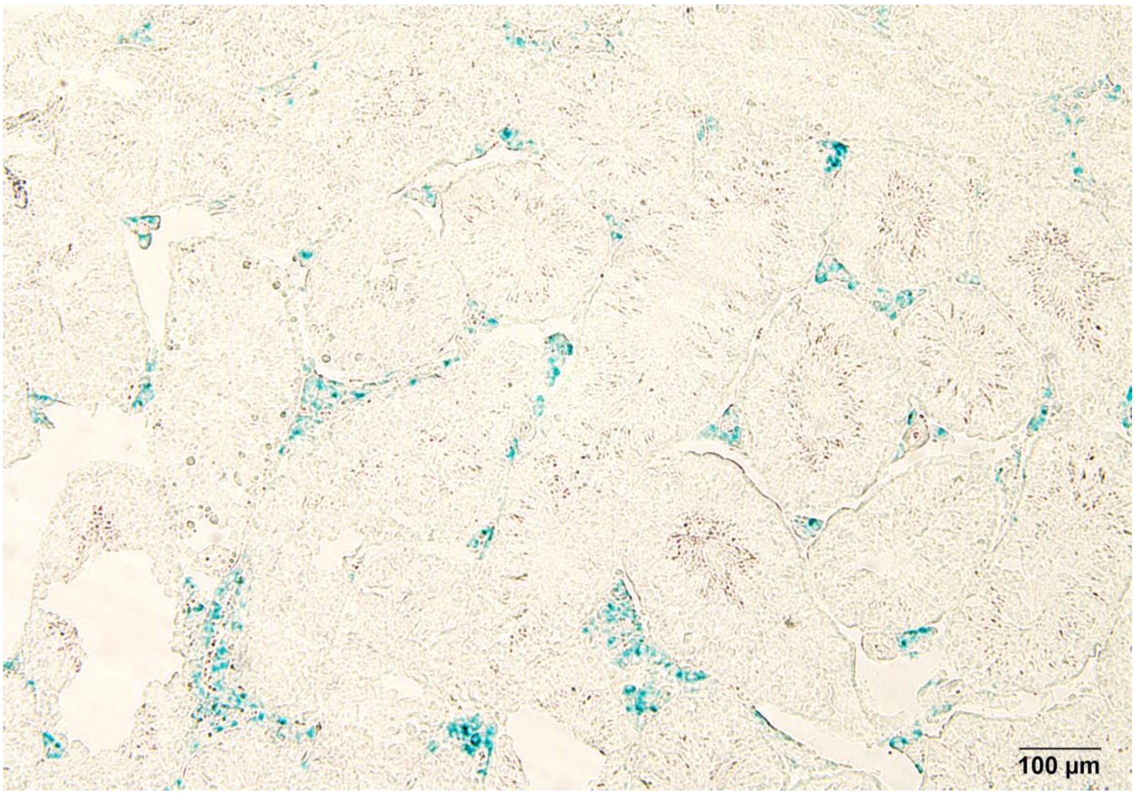

Supplementary Figure S2. PLAG1-expressing cells in adult (>2 months old) mouse testis. (**a**) *lacZ*-positive cells (blue) in the testis of a *Plag1* knock-out animal, as determined by X-gal staining with Nuclear Fast Red counterstain. (**b**) Wild-type negative control for X-gal staining showing endogenous galactosidase-like activity in the Leydig cells, but not in the tubules. Section thickness was 12 µm.

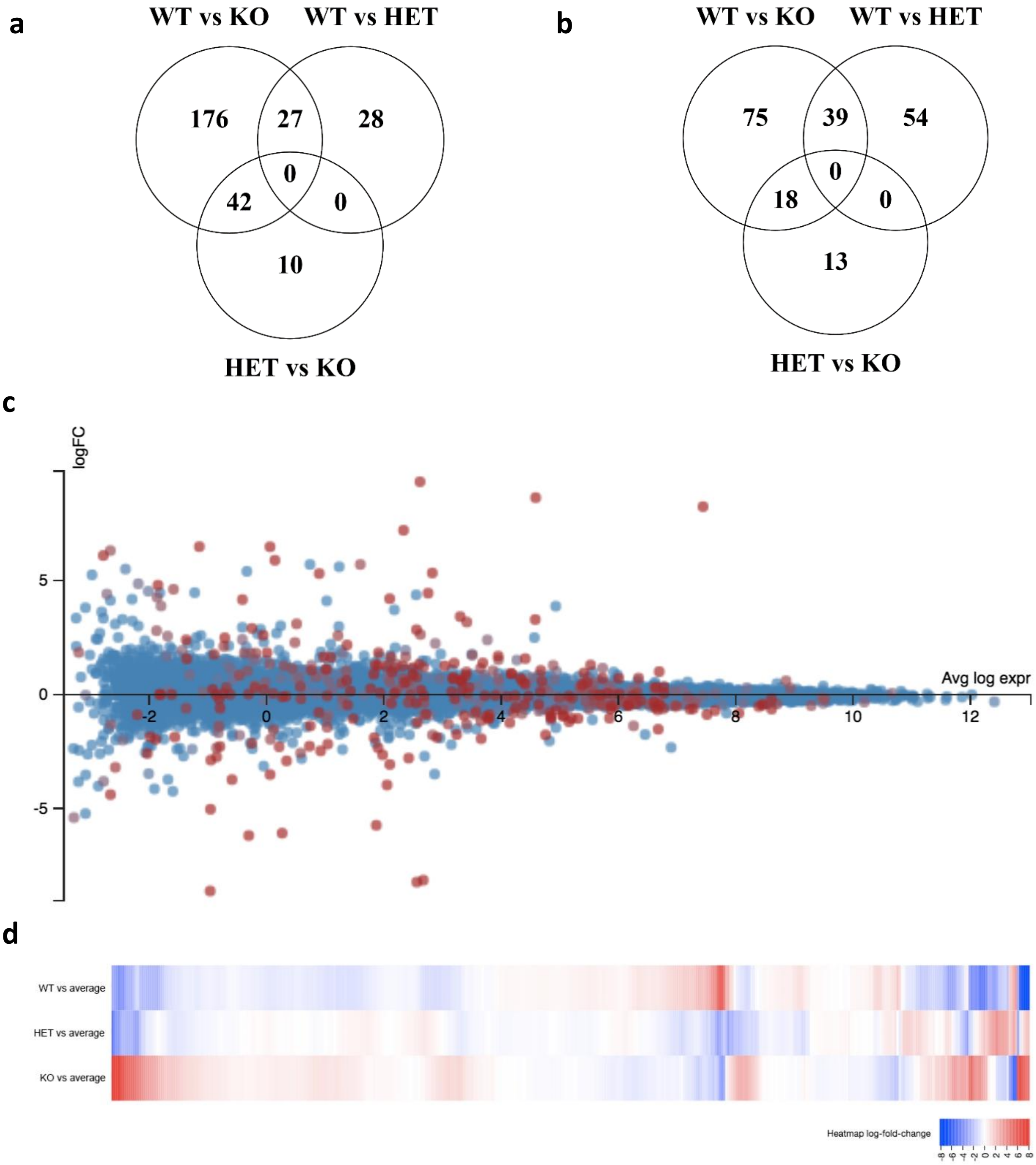

Supplementary Figure S3. Differentially expressed genes between *Plag1* wild-type (WT), heterozygous (HET), and knock-out (KO) mouse testes. (**a**) A total of 245 genes were upregulated and (**b**) 132 genes were downregulated in the KO mice compared to the WT mice. (**c**) MA plot of all 18,525 genes. An MA plot shows on a logarithmic scale the relationship between the $\log_2$ fold change for each gene and the average $\log_2$ expression. Blue dots correspond to genes that are expressed at a similar level in KO, HET and WT testes; red dots are differentially expressed genes (FDR<0.05). (**d**) Heat map comparing individual gene expression across the three genotypes, showing each individual differentially expressed gene as a specific colour based on a positive (red) or negative (blue) log fold change. Genes displayed in white have a log fold change of 0 compared to the average gene expression levels.

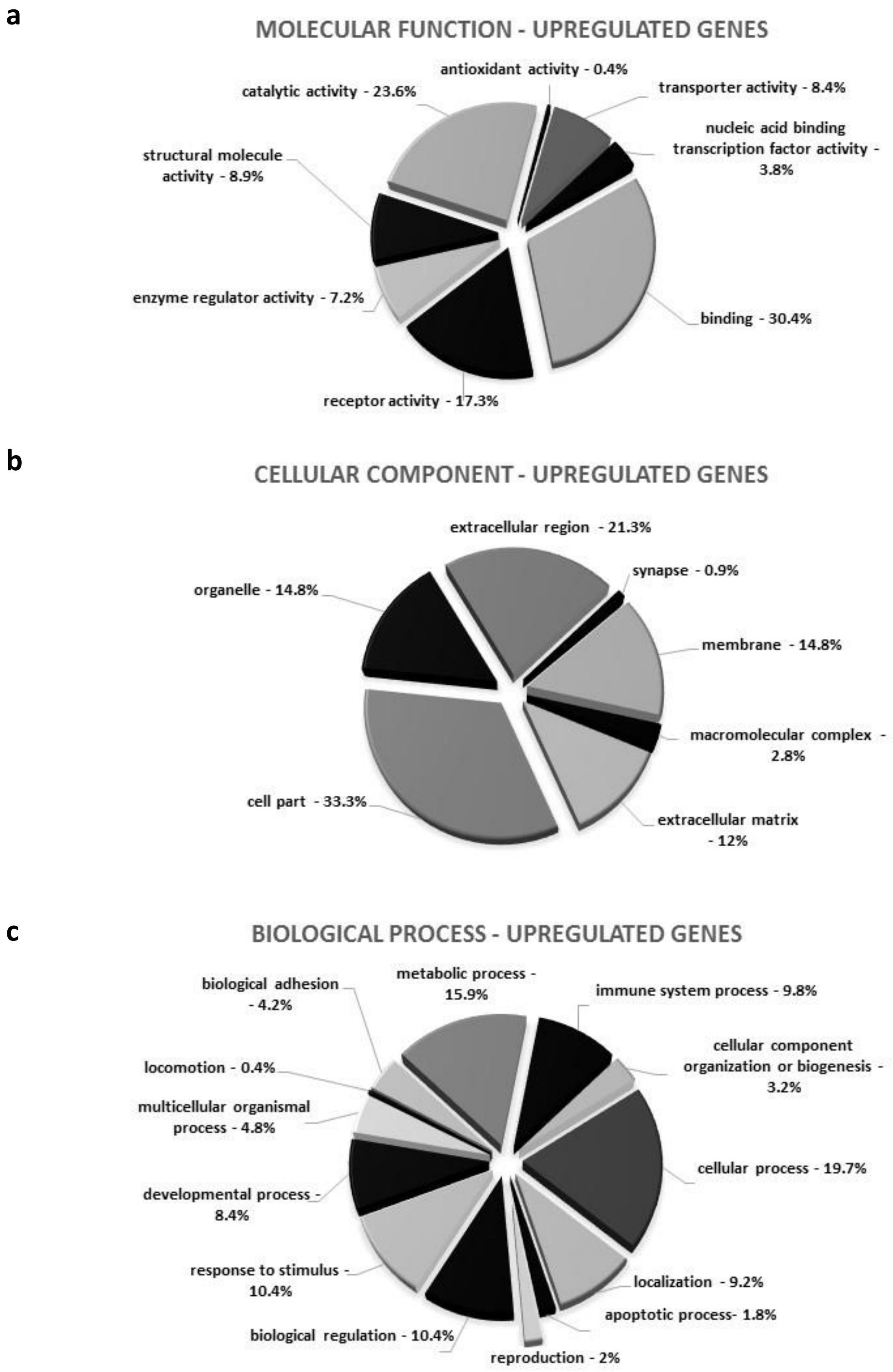

Supplementary Figure S4. Gene ontology of the upregulated and downregulated genes in *Plag1* knock-out compared to heterozygous and wild-type mouse testes. *Continued on the next page*.

d

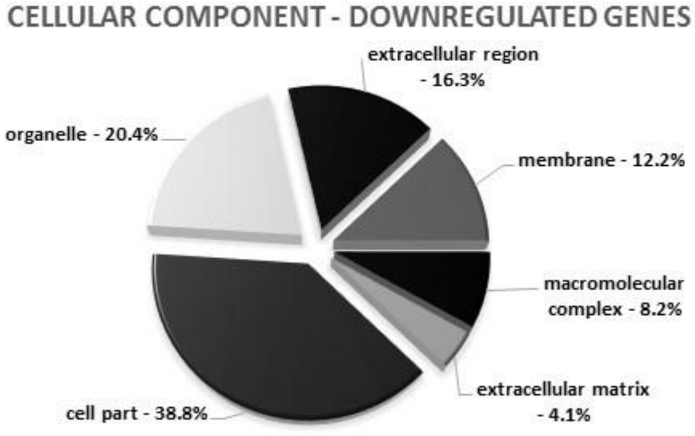

e

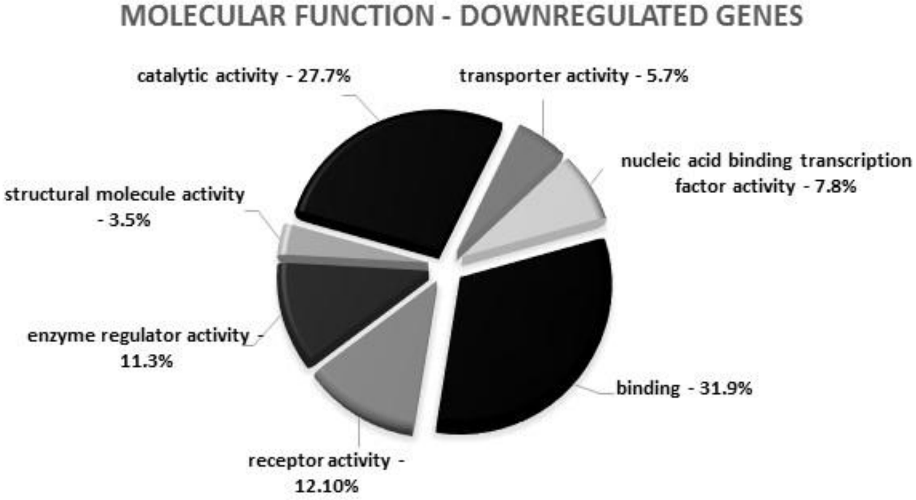

f

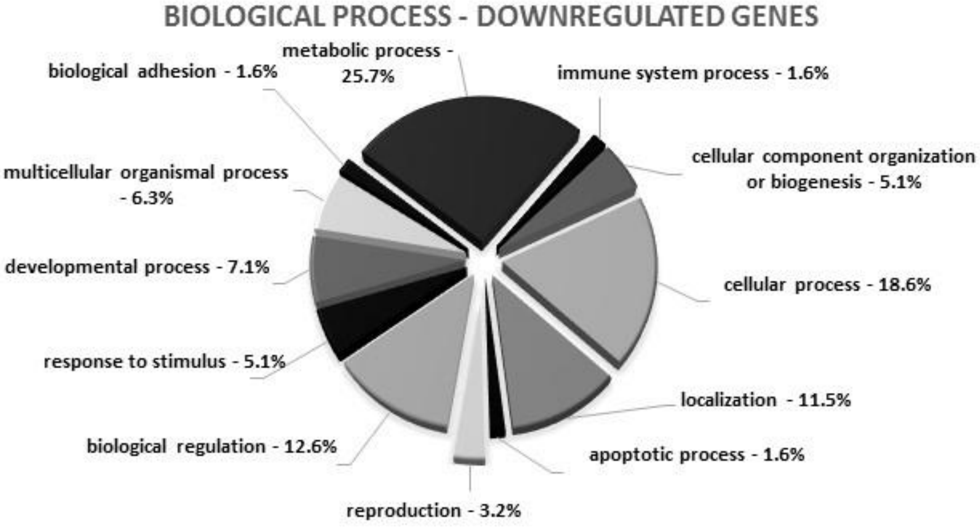

Supplementary Figure S4. Gene ontology of the upregulated and downregulated genes in *Plag1* knock-out compared to heterozygous and wild-type mouse testes. The genes were classified by (**a,d**) molecular function, (**b,e**) cellular component, and (**c,f**) biological process.

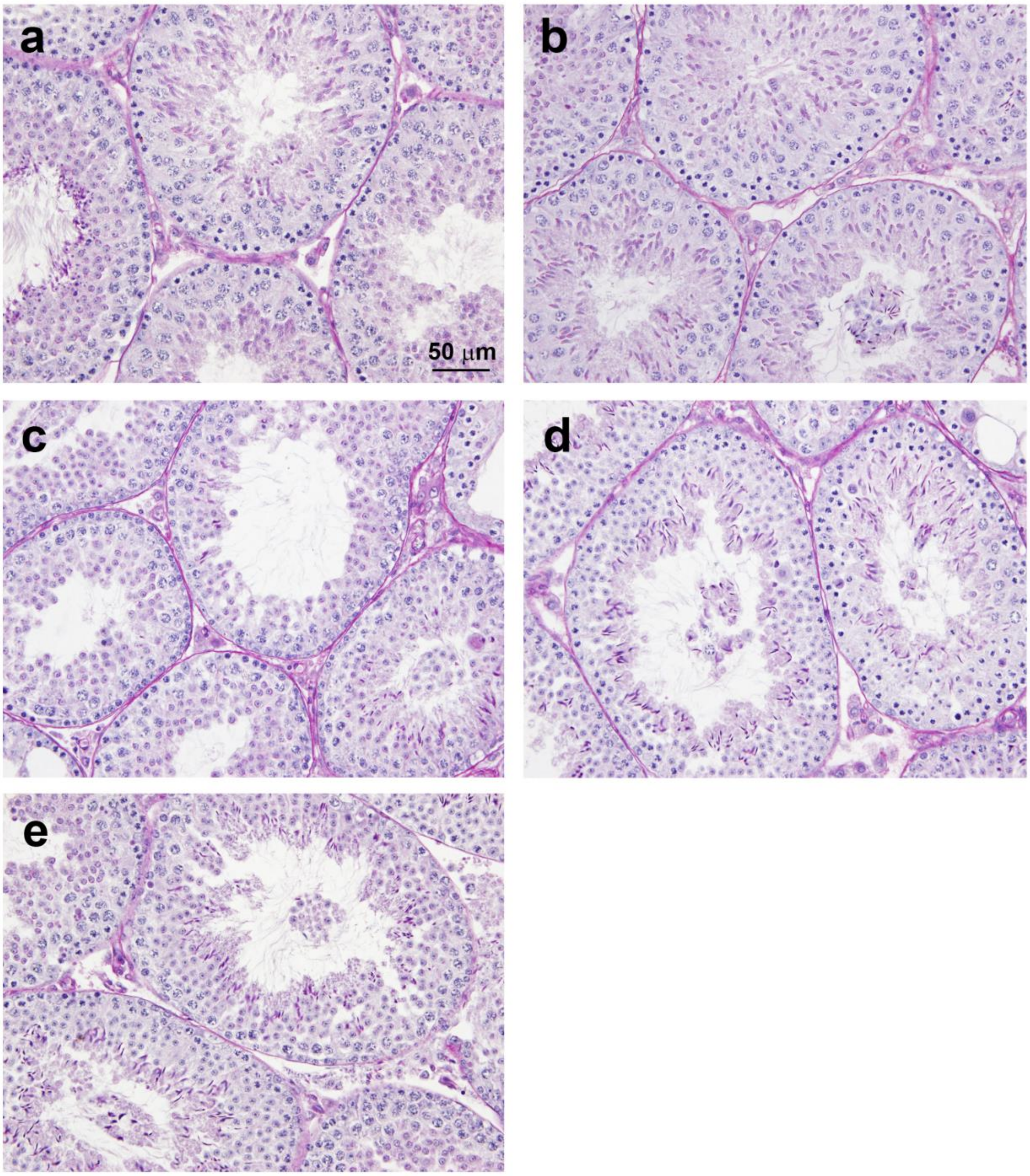

Supplementary Figure S5. Histology of the testes of 18–23-week-old mice. (**a**) Representative seminiferous tubules of a *Plag1* wild-type male. (**b**–**e**) Seminiferous tubules showing sloughing of the germinal epithelium in different *Plag1* knock-out animals. Sections were stained with periodic acid–Schiff and hematoxylin. Section thickness was 5 μm. Scale bar for all photos is as shown in panel (a).

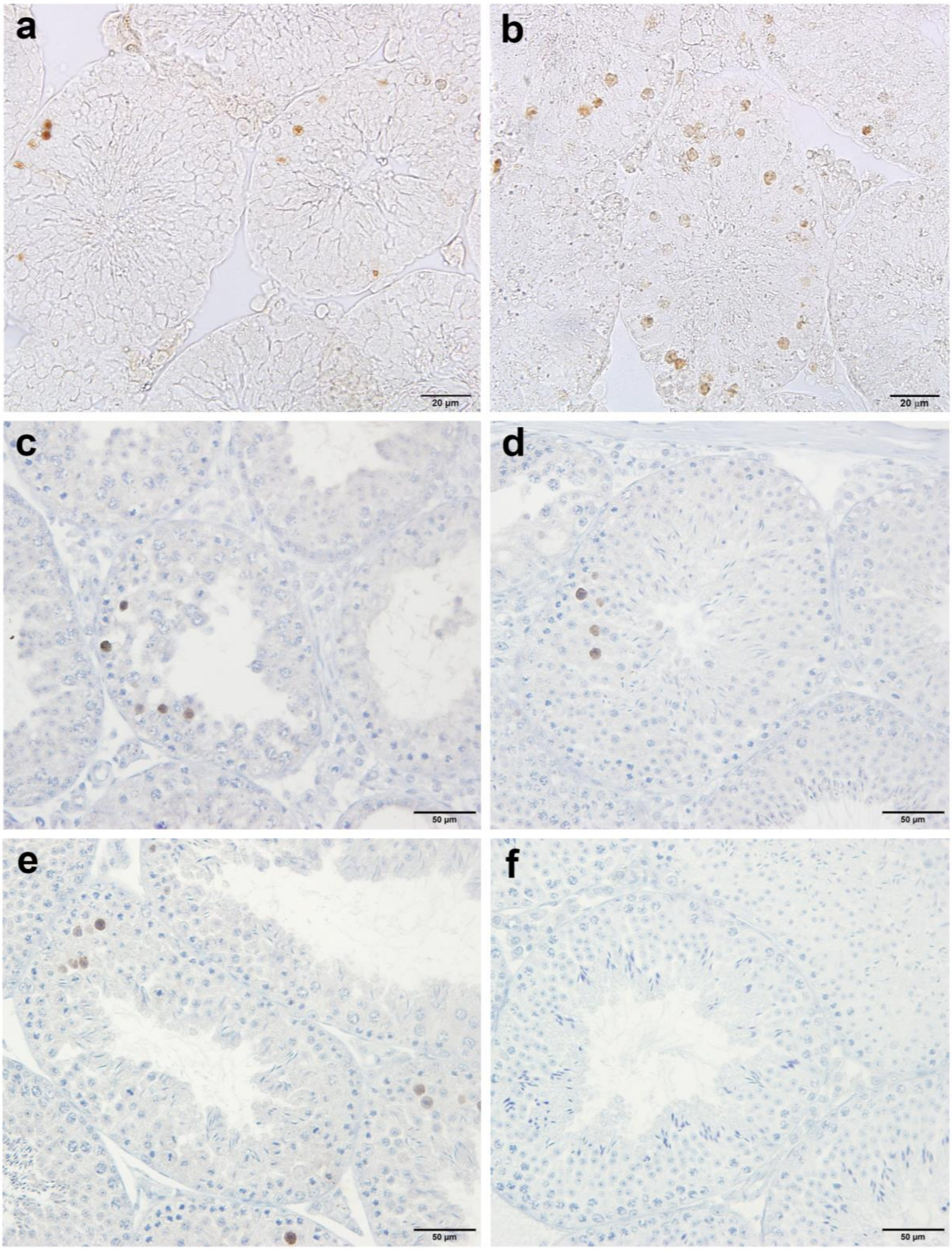

Supplementary Figure S6. Apoptotic cells in the seminiferous tubules of 5-week-old mice. TUNEL stain (**a,b**) and immunostaining for cleaved Caspase 3 and cleaved Caspase 9 (**b**–**f**) confirmed that the cells that were considered dead or dying cells in testis sections of *Plag1* wild-type and knock-out mice stained for histology were apoptotic. Sections shown here were all from knock-out mice, except (e) and (f). (f) is a negative control for the Caspase stain (omission of primary antibodies). TUNEL stain was performed using the DeadEnd Colorimetric TUNEL System (Promega, Madison, WI, USA) following the manufac-turer's guidelines. Immunostaining of Caspases was performed as described by O'Bryan *et al.*, *PLoS Genet*. 2013 9:e1003628. Section thickness was 5 µm.
